# Uniportal video-assisted thoracoscopic surgery for lobectomy: the learning curve

**DOI:** 10.1093/icvts/ivad135

**Published:** 2023-08-12

**Authors:** Iris E W G Laven, Jean H T Daemen, Aimée J P M Franssen, Michiel H M Gronenschild, Karel W E Hulsewé, Yvonne L J Vissers, Erik R de Loos

**Affiliations:** Department of Surgery, Division of General Thoracic Surgery, Zuyderland Medical Center, Heerlen, the Netherlandsds; Department of Surgery, Division of General Thoracic Surgery, Zuyderland Medical Center, Heerlen, the Netherlandsds; Department of Surgery, Division of General Thoracic Surgery, Zuyderland Medical Center, Heerlen, the Netherlandsds; Department of Respiratory Medicine, Zuyderland Medical Center, Heerlen, the Netherlands; Department of Surgery, Division of General Thoracic Surgery, Zuyderland Medical Center, Heerlen, the Netherlandsds; Department of Surgery, Division of General Thoracic Surgery, Zuyderland Medical Center, Heerlen, the Netherlandsds; Department of Surgery, Division of General Thoracic Surgery, Zuyderland Medical Center, Heerlen, the Netherlandsds

**Keywords:** Uniportal video-assisted thoracoscopic surgery lobectomy, Learning curve, Cumulative sum, Complications, Surgery duration

## Abstract

**OBJECTIVES:**

Prior reported learning curves for uniportal video-assisted thoracoscopic lobectomy were predominantly based on surgery duration, while reports on complications are limited. Therefore, our study assessed the learning curve based on both technique-related complications and surgery duration.

**METHODS:**

We retrospectively collected data from patients who had undergone uniportal video-assisted thoracoscopic lobectomy between 2015 and 2020. Exclusion criteria were concomitant procedures other than ipsilateral wedge resection, discontinued procedures, or lost to follow-up (less than 30 days). Learning curves were constructed per surgeon who performed over 20 procedures using non-risk adjusted cumulative sum (CUSUM) analysis for technique-related complications and cumulative sum analysis for surgery duration. Based on the literature, an acceptable complication rate was set at 30%, an unacceptable complication rate at 45%, and a mean surgery duration of 145 min.

**RESULTS:**

Learning curves were constructed for three thoracic surgeons and one fellow who performed 324 uniportal video-assisted thoracoscopic lobectomies in total. Each surgeon was experienced in multiportal video-assisted thoracoscopic lobectomy, the fellow was familiar with basic multiportal video-assisted thoracoscopic procedures. Cumulative sum charts of three surgeons reached a statistically significant technique-related complication rate below 30% between 50 and 96 procedures. Regarding surgery duration, typical learning curves were observed for three surgeons with a transition point between 14 and 26 procedures.

**CONCLUSIONS:**

Learning of uniportal video-assisted thoracoscopic surgery for lobectomy is safe without unacceptable complication rates and has a declining surgery duration over time for thoracic surgeons with experience in multiportal video-assisted thoracoscopic lobectomies. However, it remains unknown when the different stages of mastery are completed.

**Figure:**
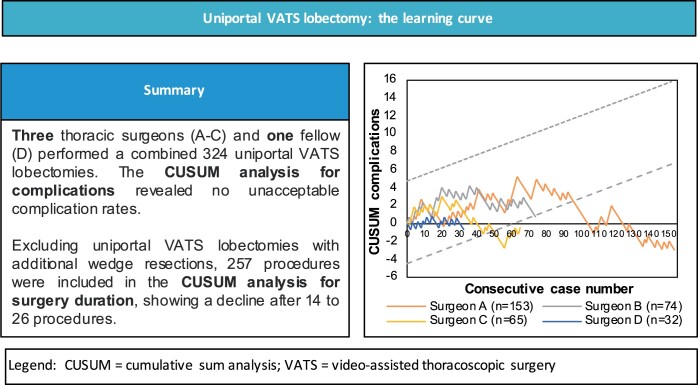

## INTRODUCTION

Initially, video-assisted thoracoscopic surgery (VATS) was performed using two to four access ports (multiportal VATS). Two decades ago, uniportal VATS made its debut in thoracic surgery [1], and in 2010, the first lobectomy was performed through a single incision [2]. Suggested advantages of uniportal over a multiportal VATS approach for anatomical resections include less intraoperative blood loss, reduced postoperative pain, and shortened hospitalization [3].

Despite these potential advantages, a European questionnaire on the clinical use of VATS reported that only 13% of the 461 surgeons from 100 responding institutions performed a lobectomy primarily via the uniportal VATS approach [4]. A possible barrier to starting a uniportal VATS program may be an associated learning curve with potentially increased complication rates during the initial learning phase.

To date, multiple centers have evaluated the learning curve of uniportal VATS lobectomy (Supplementary Material, Table S1) [5–12], predominantly based on surgery duration [5–11]. While complications associated with the learning curve unjustly received considerably less or no attention.

The learning curve of uniportal VATS lobectomy has not been evaluated for technique-related complications for multiple surgeons from the same surgical center before. Therefore, this study aimed to describe the learning process of multiple surgeons in a single center starting a uniportal VATS lobectomy program by evaluating technique-related complications alongside surgery duration.

## PATIENTS AND METHODS

### Study design, participants, and setting

**Figure 1:**
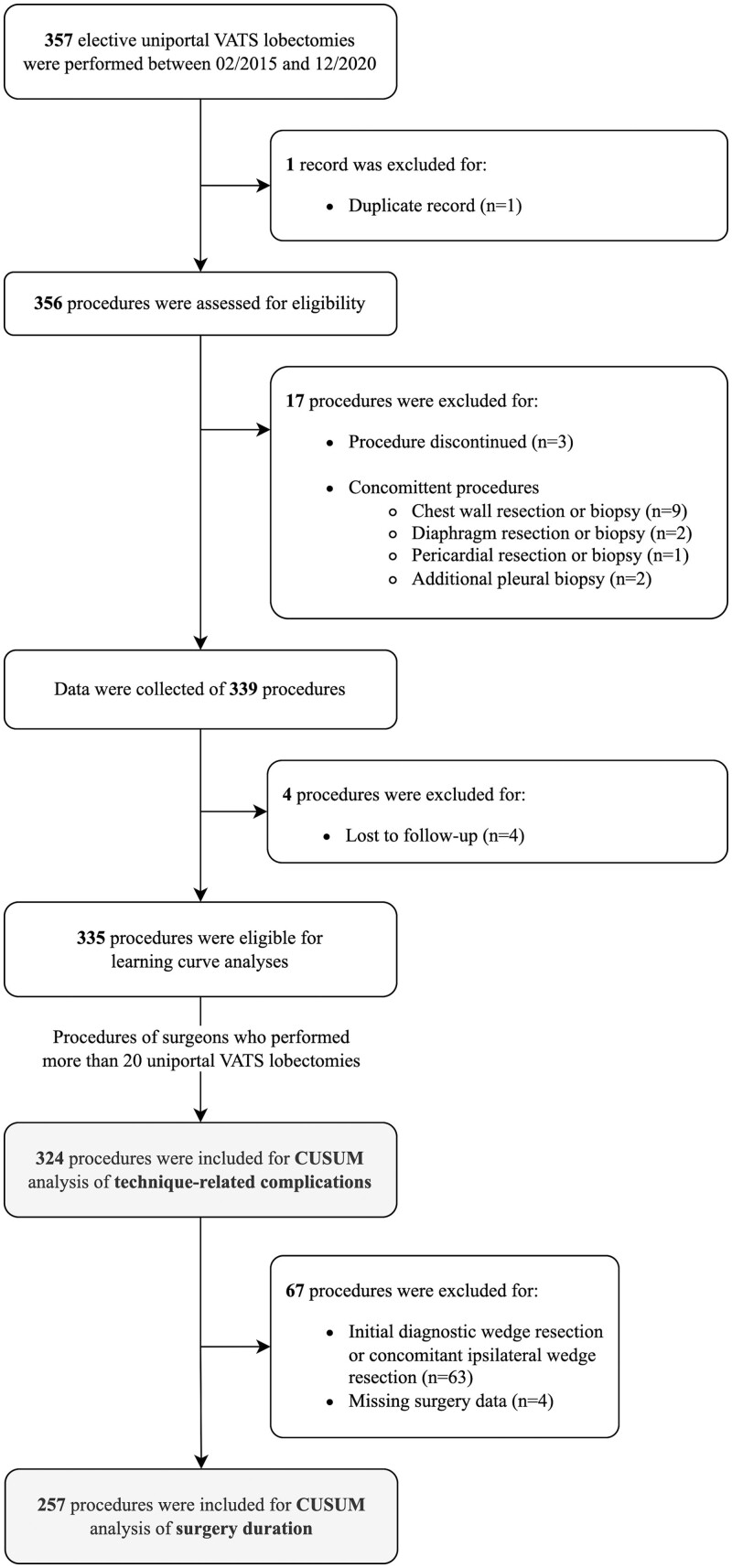
Inclusion flow chart. CUSUM: cumulative sum; VATS: video-assisted thoracoscopic surgery.

A single-center retrospective cohort study was performed at Zuyderland Medical Center (Heerlen, The Netherlands). Data of patients who had undergone a uniportal VATS lobectomy between February 2015, the start of the uniportal VATS program for lobectomy in our surgical center, and December 2020 were obtained from electronic patient records. Inclusion criteria were intention to uniportal VATS lobectomy, with or without prior wedge resection with frozen section analysis to establish a diagnosis before lobectomy. Exclusion criteria were lobectomy with a concomitant procedure (i.e. pleural biopsy or resection of the chest wall, diaphragm, or pericardium) other than an ipsilateral wedge resection, discontinued procedures (i.e. a procedure that was prematurely ended before the targeted lobe could be resected due to, e.g., severe adhesions preventing accessibility of the thoracic cavity, anesthesiologic issues, or intramural hematoma of the great vasculature), and patients whose follow-up was less than 30 days (i.e. patients who returned to their referring hospital for follow-up after the surgical procedure took place in our hospital). The included procedures were eligible for the learning curve analysis of technique-related complications. For the learning curve analysis of surgery duration, procedures were excluded in which a diagnostic wedge resection with frozen section analysis was performed before definitive lobectomy, concomitant ipsilateral wedge resection was performed, or if surgery duration data were missing. Figure 1 shows the patient inclusion chart. This study was approved by the local ethics committee of Zuyderland Medical Center, Heerlen, The Netherlands (approval number: METCZ20210021; approval date: 8 February 2021), waiving the need for individual patient consent. This report was written following the Strengthening the Reporting of Observational Studies in Epidemiology (STROBE) statement.

### Surgeons

Each procedure was performed by a thoracic surgeon assisted by another thoracic surgeon or trainee and a scrub nurse. Thoracic surgeons and trainees who performed over 20 uniportal VATS lobectomy procedures in total were included in the learning curve analyses.

### Surgical technique

Patients received general anesthesia with single-lung ventilation using a double-lumen endotracheal tube. Prophylactic cefazoline was administered intravenously. After draping, a 10mm endoscope with a 30° angle (Olympus, Hoofddorp, The Netherlands) was introduced via a 4cm incision in the midaxillary line, at the level of the 5th intercostal space. When no preoperative pathological diagnosis was available, a diagnostic wedge resection of the target lesion was performed, if possible, and analyzed by frozen section first. Subsequently, a definitive lobectomy was carried out in case of proven malignancy. Vessels, bronchi, and the fissure were transected using a linear endostapler with tissue-specific cartridges (Ethicon Endo-Surgery, Johnson&Johnson, Amersfoort, The Netherlands). If indicated (e.g. the tumor was suspected of invasion in the adjacent lobe), an additional wedge resection of an ipsilateral lobe was performed. Concurrently, lobe-specific systemic nodal dissection was performed according to international oncological guidelines [13, 14], with dissection of mediastinal tissue containing specific lymph node stations depending on the lobar location of the primary tumor. At the end of the surgery, a 28Fr chest tube was guided through the uniportal incision for pleural drainage.

### Postoperative management

For postoperative analgesia, a multi-level (T2 to T8) intercostal block, thoracic epidural catheter, or erector spinae plane block was applied. All patients received postoperative thrombosis prophylaxis and analgesia including oral nonsteroidal anti-inflammatory drugs and acetaminophen. A plain chest radiograph was acquired postoperatively. Patients were managed in a dedicated thoracic unit unless admission to the intensive care unit was required due to complications or severe cardiopulmonary morbidity. Patients were instructed by a physiotherapist to perform thoracic expansion exercises and incentive spirometry. Criteria for chest tube removal included no air leakage and a drainage volume of 450 ml/day or less. Patients were discharged when the chest drain was removed, they were mobile and oral analgesics sufficed to manage their pain.

### Variables

Demographic, clinical, and perioperative characteristics encompassed age, sex, Eastern Cooperative Oncology Group (ECOG) performance status [15], body mass index, American Society of Anesthesiologists (ASA) classification, pulmonary function parameters (forced expiratory volume in 1 s [FEV1]; diffusion capacity of the lung for carbon monoxide by the single-breath technique [DLCO-SB]), smoking history, comorbidities, tumor location, tumor diameter, preoperative video-assisted mediastinoscopic lymphadenectomy (VAMLA), presence of pleural adhesions, blood loss, conversion to either multiportal VATS or thoracotomy, and surgery duration (time between skin incision and closure).

Postoperative data were collected on chest drainage duration, postoperative length of hospital stay, technique-related and other complications, readmission, pathology, and radicality. Postoperative hospitalization was defined as the total number of days after surgery, starting from the day of surgery (index day; i.e. day zero) until the day the patient was clinically discharged from the department of thoracic surgery. Definitions of perioperative complications are provided in Supplementary Material, Table S2. Technique-related complications were defined as any intraoperative or postoperative complication within 30 days after the surgery related to the surgical technique. These included mortality, conversion, intraoperative blood loss of more than 500cc, blood transfusion during surgery or within the first two postoperative days, surgical reintervention, prolonged drainage duration (more than five days), laryngeal recurrence nerve palsy, chylothorax, and surgical site infection. Anesthesia-related complications (i.e. complications associated with ventilation, urinary catheter, or medication), complications related to the patient’s comorbidities, or other patient-related complications were defined as ‘other complications’ and not taken into account. Complications were graded according to the Clavien–Dindo classification and classified as minor (Clavien–Dindo = I–II) or major complications (Clavien–Dindo ≥III) [16]. An overview of technique-related and other complications is summarized in Supplementary Material, Table S3.

### Statistical analyses

Standard statistical analyses were performed by SPSS statistics (IBM SPSS Statistics for MacOS, Version 26.0; IBM Corp., Armonk, NY, USA). Nominal variables were denoted as frequency and percentage. Continuous variables were reported as mean and standard deviation or as median and 25th percentile (p25) and 75th percentile (p75) for non-normally distributed data. Perioperative characteristics were compared between surgeons using the chi-squared test or Fisher’s exact test (in case of frequencies less than 5) for categoric variables, the Kruskal–Wallis test for non-normal distributed continuous variables, and the one-way analysis of variance test for normally distributed continuous variables. A *P*-value of less than 0.05 was considered statistically significant. Missing data were reported as such.

### Cumulative sum analysis for technique-related complications

Non-risk adjusted cumulative sum (CUSUM) observed minus expected failure was used to assess technique-related complications of patients who underwent a lobectomy with or without an initial diagnostic or concomitant ipsilateral wedge resection. These were constructed by Microsoft Excel (Version 2022, Microsoft Corp., Redmond, WA, USA). Statistical principles of the unadjusted cumulative failure charts were adapted from Rogers *et al.* [17] and de Loos *et al.* [18]. The CUSUM chart was incremented by 1 minus *p*_0_ (acceptable complication rate) for every failed procedure and by *p*_0_ for every successful procedure. Failure was defined as a patient experiencing any technique-related complication within 30 days after surgery and success was defined as the absence of any technique-related complications. An acceptable failure rate for technique-related complications was set at 30%, based on previously published data of uniportal VATS lobectomy cohorts (Supplementary Material, Table S4) [6, 10–12, 19–27]. The unacceptable failure rate (*p*_1_) was set at 1.5 times *p*_0_. Crossing the upper 95% boundary indicated a significant increase from *p*_0_ to *p*_1_. If the lower 95% boundary was crossed, a statistically significant failure rate equal to or lower than *p*_0_ was indicated. The chart was reset if it crossed the lower 95% boundary to prevent a build-up of credit that could mask an increase in the level of unacceptable failure.

### Cumulative sum analysis for surgery duration

Surgeon-specific CUSUM learning curves for surgery duration were constructed based on patients who had undergone a lobectomy without diagnostic or concomitant ipsilateral wedge resection. If the surgery duration of the procedure exceeded the literature-based mean surgery duration of 154 min (Supplementary Material, Table S4) [4, 8–10, 15–17, 19–23], the CUSUM chart was incremented by 1. If the surgery duration was 154 min or less, the chart was decremented by 1.

### Sensitivity analysis

CUSUM learning curves may be affected by the frequency of performed lobectomy procedures per surgeon per time period. Therefore, a sensitivity analysis was performed to determine the average time between consecutive uniportal lobectomy procedures per surgeon.

## RESULTS

Of the 357 patients who underwent a uniportal VATS lobectomy with or without initial diagnostic or concomitant ipsilateral wedge resection, 335 patients were eligible (Fig. 1). In total, 22 procedures were excluded because of discontinued procedures (*n* = 3), concomitant procedures (*n* = 14), or patients who were lost to follow-up (*n* = 4). During the enrolment period, four general thoracic surgeons, two fellows in thoracic surgery, and four thoracic surgical residents performed 335 uniportal VATS lobectomies. Three surgeons (surgeons A, B, and C) and one fellow (surgeon D) performed over 20 uniportal VATS lobectomies, with a total of 324 procedures.

### Surgeons

Surgeons A, B, and C learned the uniportal VATS technique via courses by an experienced surgeon in uniportal VATS lobectomy, wet lab sessions on animal models, annual follow-up courses or master classes, and visiting high-volume centers. Surgeon D was trained in our certified general thoracic teaching institution. Surgeon A had four years of multiportal VATS lobectomy experience, surgeon B five years and surgeon C three years, corresponding with approximately160, 100, and 60 procedures, respectively. Surgeon D had limited experience with basic multiportal VATS procedures.

### Baseline characteristics and perioperative outcomes

Table 1 summarizes the baseline characteristics of 324 patients who were included in the learning curve analyses. No significant differences in baseline characteristics were found between the surgeons (Table 1).

**Table 1:** Baseline patient characteristics

	All procedures (*n* = 324)	Missing (%)	Surgeon A (*n* = 153)	Missing (%)	Surgeon B (*n* = 74)	Missing (%)	Surgeon C (*n* = 65)	Missing (%)	Surgeon D (*n* = 32)	Missing (%)	*P*-Value
Age (years), median (p25; p75)	68.0 (62.0; 74.0)		68.0 (61.0; 74.0)		68.5 (62.0; 74.0)		67.0 (61.5; 71.5)		69.0 (65.3; 75.8)		0.162
Sex (male), *n* (%)	175 (54.0%)		76 (49.7%)		42 (56.8%)		39 (60.0%)		18 (56.3%)		0.496
ECOG (≥2), *n* (%)	41 (12.6%)		23 (15.0%)		5 (6.8%)		9 (13.8%)		4 (12.5%)		0.470
BMI (kg/m), median (p25; p75)	26.0 (23.3; 29.4)		25.6 (23.2; 28.8)		26.6 (23.3; 30.1)		25.7 (22.6; 29.5)		26.6 (23.7; 29.3)		0.509
ASA class, *n* (%)											0.155
2	12 (3.7%)		4 (2.6%)		4 (5.4%)		3 (4.6%)		1 (3.1%)		
3	310 (95.7%)		149 (97.4%)		70 (94.6%)		60 (92.3%)		30 (93.8%)		
4	2 (0.6%)		0 (0%)		0 (0%)		2 (3.1%)		0 (0%)		
FEV1 (predicted %), mean ± SD	86.1 ± 21.0		85.5 ± 21.2		88.0 ± 20.2		86.6 ± 21.6		84.0 ± 21.0		0.784
DLCO SB (predicted %), mean ± SD	76.9 ± 18.2	13 (3.9%)	76.2 ± 19.6	4 (2.6%)	76.8 ± 16.8	5 (6.8%)	76.6 ± 18.0	2 (3.1%)	81.1 ± 17.4	1 (3.1%)	0.617
Smoking history, *n* (%)	274 (84.6%)		129 (84.3%)		63 (85.1%)		52 (80.0%)		30 (93.8%)		0.372
Comorbidities, *n* (%)											
Cardiac	88 (27.2%)		40 (26.1%)		27 (36.5%)		14 (21.5%)		7 (21.9%)		0.185
Hypertension	141 (43.5%)		66 (43.1%)		33 (44.6%)		28 (43.1%)		14 (43.8%)		0.997
Diabetes mellitus	51 (15.7%)		22 (14.4%)		16 (21.6%)		8 (12.3%)		5 (15.6%)		0.437
ILD	7 (2.2%)		2 (1.3%)		1 (1.4%)		3 (4.6%)		1 (3.1%)		0.432
Malignancy	103 (31.8%)		45 (29.4%)		24 (32.4%)		24 (36.9%)		10 (31.3%)		0.751
Thoracic radiotherapy	12 (3.7%)		4 (2.6%)		4 (5.4%)		4 (6.2%)		0 (0%)		0.329
Previous lung surgery	21 (6.5%)		13 (8.5%)		6 (8.1%)		2 (3.1%)		0 (0%)		0.186
cTNM stage , *n* (%)		3 (0.9%)		1 (0.1%)		2 (2.8%)					0.297
Occult cancer	13 (4.0%)		8 (5.2%)		3 (4.1%)		2 (3.1%)		0 (0.0%)		
IA	150 (46.3%)		66 (43.1%)		32 (43.2%)		33 (50.8%)		19 (59.4%)		
IB	41 (12.7%)		24 (15.7%)		7 (9.5%)		8 (12.3%)		2 (6.3%)		
IIA	21 (6.5%)		9 (5.9%)		7 (9.5%)		4 (6.2%)		1 (3.1%)		
IIB	57 (17.6%)		30 (19.6%)		16 (21.6%)		7 (10.8%)		4 (12.5%)		
IIIA	22 (6.8%)		8 (5.2%)		6 (8.1%)		5 (7.7%)		3 (9.4%)		
IIIB	2 (0.6%)		2 (1.3%)		0 (0.0%)		0 (0.0%)		0 (0.0%)		
IVA	12 (3.7%)		5 (3.3%)		0 (0.0%)		5 (7.7%)		2 (6.3%)		
IVB	3 (0.9%)		0 (0.0%)		1 (1.4%)		1 (1.5%)		1 (3.1%)		
Location lobectomy, *n* (%)											0.381
Right upper lobe	92 (28.4%)		41 (26.8%)		22 (29.7%)		19 (29.2%)		10 (31.3%)		
Right middle lobe	17 (5.2%)		10 (6.5%)		4 (5.4%)		1 (1.5%)		2 (6.3%)		
Right lower lobe	60 (18.5%)		28 (18.3%)		17 (23.0%)		11 (16.9%)		4 (12.5%)		
Left upper lobe	89 (27.5%)		38 (24.8%)		18 (24.3%)		26 (40.0%)		7 (21.9%)		
Left lower lobe	66 (20.4%)		36 (23.5%)		13 (17.6%)		8 (12.3%)		9 (28.1%)		
Tumor diameter (mm), median (p25; p75)	25.0 (17.0; 40.0)	7 (2.1%)	25.0 (18.0; 44.3)	3 (2.0%)	25.0 (16.0; 44.0)	3 (4.1%)	21.5 (15.3; 35.0)	1 (1.5%)	24.5 (18.0; 34.8)		0.788
VAMLA, *n* (%)	128 (39.5%)		62 (40.5%)		30 (40.5%)		25 (38.5%)		11 (34.4%)		0.923
Final pathology, *n* (%)											0.918
Primary lung cancer	276 (85.2%)		130 (85.0%)		63 (85.1%)		55 (84.6%)		28 (87.5%)		
Metastasis	27 (8.3%)		14 (9.2%)		5 (6.8%)		6 (9.2%)		2 (6.3%)		
Benign or ILD	21 (6.5%)		9 (5.9%)		6 (8.1%)		4 (6.2%)		2 (6.3%)		
pTNM stage, *n* (%)		7 (2.2%)		2 (1.3%)		3 (4.1%)		2 (3.1%)			0.705
Occult cancer	47 (14.5%)		23 (15.0%)		11 (14.9%)		9 (13.8%)		4 (12.5%)		
IA	109 (33.6%)		49 (32.0%)		23 (31.1%)		26 (40.0%)		11 (34.4%)		
IB	45 (13.9%)		19 (12.4%)		8 (10.8%)		10 (15.4%)		8 (25.0%)		
IIA	21 (6.5%)		11 (7.2%)		6 (8.1%)		3 (4.6%)		1 (3.1%)		
IIB	59 (18.2%)		35 (22.9%)		15 (20.3%)		6 (9.2%)		3 (9.4%)		
IIIA	23 (7.1%)		9 (5.9%)		7 (9.5%)		5 (7.7%)		2 (6.3%)		
IIIB	3 (0.9%)		1 (0.7%)		0 (0.0%)		2 (3.1%)		0 (0.0%)		
IVA	6 (1.9%)		2 (1.3%)		1 (1.4%)		1 (1.5%)		2 (6.3%)		
IVB	4 (1.2%)		2 (1.3%)		0 (0.0%)		1 (1.5%)		1 (3.1%)		

ASA: American Society of Anesthesiologists; BMI: body mass index; DLCO SB: diffusing lung capacity single-breath; ECOG: Eastern Cooperative Oncology Group performance status; FEV1: forced expiratory volume in 1 s; ILD: interstitial lung disease; VAMLA: video-assisted mediastinoscopic lymphadenectomy

Intra- and postoperative characteristics are provided in Table 2. In total, 94 procedures were complicated due to the occurrence of at least one technique-related complication (29.0%). Fifteen procedures resulted in more than 500 cc blood loss (4.6%) and 40 conversions were required (12.3%) due to bleeding (*n* = 13) and technical difficulty (*n* = 27, e.g. severe adhesions or incomplete deflated lungs). Sixteen reoperations were performed for empyema (*n* = 10), pleural effusion (*n* = 1), postoperative bleeding (*n* = 2), prolonged air leakage (*n* = 2), or bronchopleural fistula (*n* = 1). Seven chest tube reinsertions and three blowhole incisions were performed using negative pressure wound therapy for subcutaneous emphysema (*n* = 6), pleural effusion (*n* = 1), or pneumothorax (*n* = 3). No significant differences were found in technique-related complications or other complications between the surgeons (Table 2).

**Table 2:** Perioperative outcomes

	All procedures (*n* = 324)	Missing (%)	Surgeon A (*n* = 153)	Missing (%)	Surgeon B (*n* = 74)	Missing (%)	Surgeon C (*n* = 65)	Missing (%)	Surgeon D (*n* = 32)	Missing (%)	*P*-Value
Surgery duration (min), median (p25; p75)	138.5 (113.0; 174.5)	4 (1.2%)	137.0 (114.5; 167.0)		135.0 (107.5; 173.5)	1 (1.4%)	148.0 (109.0; 187.0)	2 (3.1%)	164.0 (127.0; 183.0)	1 (3.1%)	0.450
Pleural adhesions, *n* (%)	131 (40.4%)		69 (45.1%)		27 (36.5%)		24 (36.9%)		11 (34.4%)		0.362
Blood loss (cc), median (p25; p75)	100.0 (50.0; 200.0)	9 (2.8%)	100.0 (50.0; 200.0)	3 (2.0%)	150.0 (100.0; 300.0)	3 (4.1%)	100.0 (50.0; 200.0)	1 (1.5%)	50.0 (50.0; 200.0)	2 (6.3%)	0.225
Radicality (R0 ), *n* (%)	298 (92.0%)	7 (2.2%)	143 (93.5%)	1 (0.7%)	66 (93.0%)	3 (4.1%)	60 (96.8%)	3 (4.6%)	29 (90.6%)	0	0.536
Technique-related complications, *n* (%)	94 (29.0%)		43 (28.1%)		23 (31.1%)		19 (29.2%)		9 (28.1%)		0.973
Conversion to multiportal VATS	24 (7.4%)		8 (5.2%)		8 (10.8%)		5 (7.7%)		3 (9.4%)		
Conversion to thoracotomy	16 (4.9%)		7 (4.6%)		5 (6.8%)		3 (4.6%)		1 (3.1%)		
Intraoperative bleeding >500 cc due to a surgical failure	15 (4.6%)		6 (3.9%)		2 (2.7%)		5 (7.7%)		2 (6.3%)		
Major complications (C–D ≥ III), *n* (%)											
Chest tube reinsertion or negative pressure wound therapy (IIIa)	10 (3.1%)		5 (3.3%)		2 (2.7%)		2 (3.1%)		1 (3.1%)		
Reoperation (IIIb)	16 (4.9%)		5 (3.3%)		4 (5.4%)		5 (7.7%)		2 (6.3%)		
Mortality (V)	3 (0.9%)		2 (1.3%)		1 (1.4%)		0 (0%)		0 (0%)		
Minor complications (C–D = I–II), *n* (%)											
Prolonged drainage >5 days (I)	47 (14.5%)		27 (17.6%)		8 (10.8%)		9 (13.8%)		3 (9.4%)		
Blood transfusion (II)	4 (1.2%)		1 (0.7%)		0 (0%)		1 (1.5%)		2 (6.3%)		
Wound infection (II)	1 (0.3%)		0 (0%)		0 (0%)		1 (1.5%)		0 (0%)		
Recurrent laryngeal nerve palsy (II)	1 (0.3%)		0 (0%)		1 (1.4%)		0 (0%)		0 (0%)		
Chylothorax (II)	1 (0.3%)		1 (0.7%)		0 (0%)		0 (0%)		0 (0%)		
Other complications (%, C–D = I–II), *n* (%)	68 (21.0%)		34 (22.2%)		14 (18.9%)		11 (16.9%)		9 (28.1%)		0.580
Postoperative length of hospital stay (days), median (p25; p75)	5.0 (3.0; 8.0)		5.0 (3.0; 9.0)		4.0 (3.0; 8.0)		5.0 (3.0; 8.0)		5.0 (3.3; 7.0)		0.183
Chest drainage duration (days), median (p25; p75)	2.0 (1.0; 3.0)		2.0 (1.0; 4.0)		2.0 (1.0; 3.0)		2.0 (1.0; 3.0)		1.0 (1.0; 2.0)		0.111

C–D: Clavien–Dindo classification; VATS: video-assisted thoracoscopic surgery.

**Figure 2:**
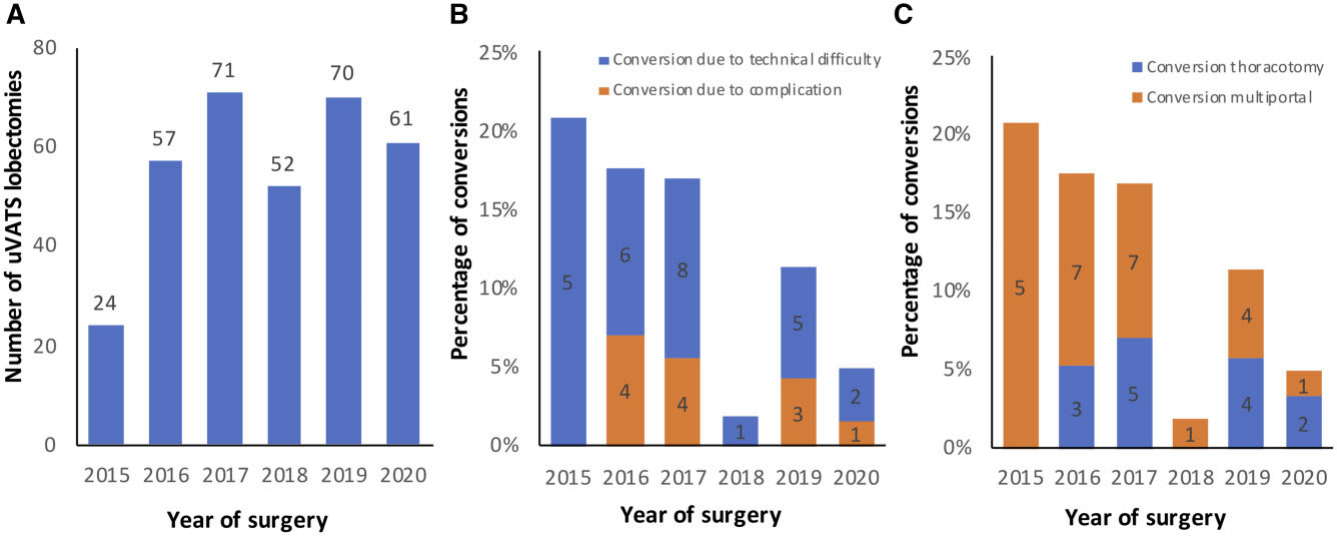
(**A**) Number of uniportal VATS lobectomies per year. (**B**) Conversion percentage due to complications or technical difficulty per year. (**C**) Conversion percentage into multiportal VATS or thoracotomy per year. VATS: video-assisted thoracoscopic surgery.

Figure 2A shows the annual number of uniportal VATS lobectomies during the enrolment period. Over the years, the annual conversion percentage had a decreasing trend from 20% to 5% (Fig. 2B and C).

### Cumulative sum analysis for technique-related complications

**Figure 3:**
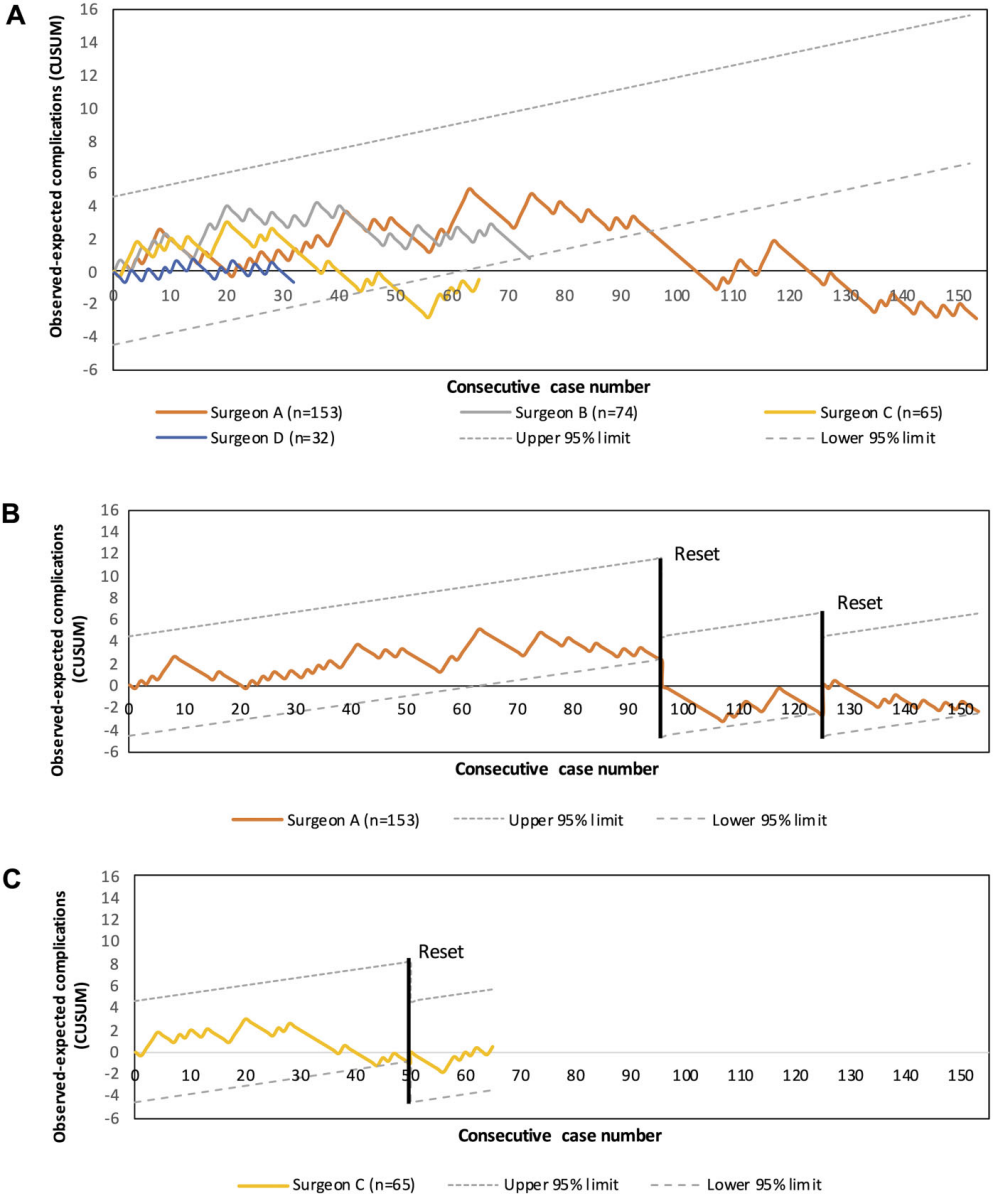
Non-risk adjusted observed minus expected CUSUM charts of technique-related complications with 95% boundary limits of patients who had undergone uniportal VATS lobectomy using an acceptable and unacceptable failure rate of 30% and 45%, respectively, for (**A**) 4 individual surgeons. CUSUM curves were reset to 0 for (**B**) surgeon A after 96 and 125 cases and (**C**) surgeon C after 50 cases. CUSUM: cumulative sum; VATS: video-assisted thoracoscopic surgery.

In total, the three surgeons and one fellow performed 324 uniportal VATS lobectomies. One hundred fifty-three uniportal VATS lobectomies with or without wedge resection were performed by surgeon A, 74 by surgeon B, 65 by surgeon C, and 32 by surgeon D. Figure 3 illustrates the constructed CUSUM charts of technique-related complications per surgeon. The charts of surgeons A, B, and C crossed the lower 95% boundary limit after 96 cases, 74 cases, and 50 cases, respectively, indicating a statistically significant technique-related complication rate of less than 30%. CUSUM charts and boundary lines were reset for surgeons A (Fig. 3A) and C (Fig. 3B), after which the CUSUM charts decreased steadily.

### Cumulative sum analysis for surgery duration

**Figure 4:**
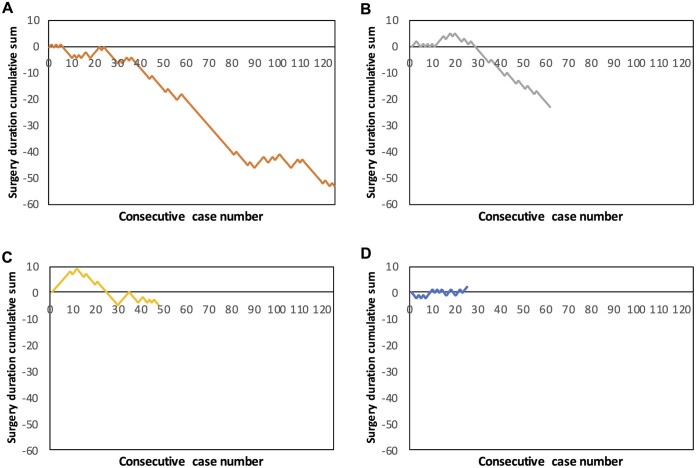
CUSUM curves for surgery duration of patients who had undergone uniportal VATS lobectomy for (**A**) surgeon A (*n* = 125), (**B**) surgeon B (*n* = 61), (**C**) surgeon C (*n* = 47), and (**D**) surgeon D (*n* = 24). CUSUM: cumulative sum; VATS: video-assisted thoracoscopic surgery.

In total, 263 of the 335 eligible procedures (78.5%) were lobectomies without diagnostic or concomitant ipsilateral wedge resection, of which 257 procedures were performed by surgeons A–D. Data on surgery duration were missing for four patients (Table 2). The median surgery duration was 135 min (p25 = 110.0; p75 = 170.0). Surgeon A (*n* = 125; 136.0 min, p25 = 110.0; p75 = 164.0), B (*n* = 61; 129.0 min, p25 = 106.0; p75 = 163.5), and C (*n* = 47; 142.0 min, p25 = 109.0; p75 = 187.0) demonstrated a typical learning curve, characterized by an initial incline followed by a horizontal plateau whereafter a decline with increasing experience was observed (Fig. 4A–C). The change in slope to a decline in surgery duration was present between 14 and 26 cases for surgeons A, B, and C. Surgeon D (*n* = 24; 162.5 min, p25 = 124.8; p75 = 180.5) showed an increase in CUSUM chart for surgery duration (Fig. 4D).

### Sensitivity analysis

Sensitivity analysis demonstrated a median number of nine days (p25 = 2; p75 = 19) between the procedures for surgeon A, 15 days for surgeon B (p25 = 7; p75 = 35), 19 days for surgeon C (p25 = 7; p75 = 44) and seven days for surgeon D (p25 = 1; p75 = 11).

## DISCUSSION

Our single-centre retrospective cohort study evaluated the learning process of uniportal VATS lobectomy using CUSUM learning curves for technique-related complications and surgery duration. Four surgeons performed between 32 and 153 uniportal VATS lobectomies individually. In total, 29.0% of the included patients experienced at least one technique-related complication. Cumulative sum charts of technique-related complications showed that the acceptable complication rate boundary line was crossed for three surgeons, indicating a statistically significant technique-related complication rate of less than 30%. Regarding surgery duration, surgeons performed between 24 and 125 uniportal VATS lobectomies without a diagnostic or concomitant wedge resection. Three experienced surgeons showed a typical learning curve in which surgery duration decreased after 14–26 procedures, while the fellow showed an increase over time.

So far, only Zhai *et al.* [12] reported a complication-based learning curve. Our reported number of cases required before a significant decrease in technique-related complications is shown (between 50 and 96 procedures), is in agreement with the 61 procedures as the initial learning phase of a single surgeon with limited open lobectomy experience published by Zhai *et al.* [12]. Whereas Zhai *et al.* performed a learning curve on surgical failures which also included pneumonia, cardiac complications, subcutaneous emphysema, delirium, less than 10 harvested lymph nodes , and 30-day readmissions, we performed a complication-based learning curve on predefined technique-related complications. Additionally, they constructed a risk-adjusted CUSUM and did not use any boundary lines indicating acceptable or unacceptable complication rates. Since there is no validated risk scoring system in the literature available for uniportal VATS lobectomies on perioperative complications, we did not perform a risk-adjusted CUSUM learning curve analysis.

Our learning curves on surgery duration demonstrated a decline after 14 to 26 procedures, which is in line with the previously reported number of procedures in the initial learning phase varying between 19 and 60 procedures (Supplementary Material, Table S1) [5–12]. Our CUSUM analysis for surgery duration varied from the one which is mostly applied in literature, whereas the chart is incremented or decremented by the individual positive or negative difference with the mean of all data points of the procedures in chronological order [5–9, 11, 12]. Our analysis prevents a disproportionally large influence of relatively extreme prolonged surgery durations (e.g., complicated surgery, more complex procedures) on the surgeon’s learning curve. However, it should be noted that the mean surgery duration threshold of 154 min in our CUSUM analysis was derived from worldwide uniportal VATS cohorts with heterogeneous differences in patient selection (e.g., upper and lower lobectomy) and surgical technique (e.g. inclusion or exclusion of the duration of lymphadenectomy). In addition, 39% of our patient population had undergone a VAMLA prior to the surgery, which may have resulted in shortened duration of the surgery since lymph node stations 2R/L, 4R/L, and 7 do not have to be intraoperatively removed anymore. This potentially could have led to an underestimation of the number of procedures until the surgery duration declines.

Surgeon D, a thoracic surgical fellow, did not show a typical learning curve for surgery duration and showed a prolonged surgery duration over time, possibly because the surgeon was still in his initial learning phase and might have been increasingly confronted with more difficult operations and less selective patient selection. This was confirmed by Wu *et al.* [11], who estimated that junior consultants should be able to be competent in uniportal VATS lobectomies after 30 procedures if they had taken a systematic training course before the start of performing uniportal VATS lobectomies. Therefore, we cannot indicate the number of cases to achieve a transition point in learning for a fellow with limited prior multiportal VATS experience. This should be analyzed further in future studies.

Several factors should be considered when extrapolating these results to other surgical departments planning to learn uniportal VATS lobectomy. First, three surgeons had between three and five years of multiportal VATS experience before the start of their first uniportal VATS lobectomy, while the fellow only had limited multiportal VATS experience with basic procedures. Additionally, all procedures were assisted by another surgeon or a trainee.

No statements can be made about the number of procedures required to achieve a certain level of mastery (novice to expert) based on complications. Li and colleagues [6] reported the number of procedures required to achieve four stages of proficiency based on surgery duration, including 52 procedures to complete the preliminary learning stage, 156 procedures for the preliminary proficiency stage, 244 procedures for the proficiency stage, and 244 for the proficiency stage. However, we believe that the level of competence should primarily be based on the number of complications, rather than on surgery duration alone.

Our study has important drawbacks. First, the study's retrospective design and inherent bias due to missing data. However, we believe our analyses were minimally affected because only four patients lacked data on surgery duration. Second, our acceptable and unacceptable complication rates were based on our definition of a technical complication related to the surgery. Because we have included conversions to multiportal VATS as a technical complication, our acceptable and unacceptable complication rate may therefore seem higher than expected. Third, the learning curve is affected by several factors which were inevitable to exclude, such as the experience gained from performing other uniportal VATS pulmonary resections during the enrolment period, as well as the multiportal VATS lobectomies performed during the transition period. Nevertheless, the selection bias on the difficulty of lobectomy procedures was limited since only 63 elective multiportal VATS lobectomies were performed during the study period, and 47 of these procedures were performed within the first year (2015), indicating a short transition period to uniportal VATS. Fourth, surgery duration varies for upper or lower lobectomy, the extent of intraoperative lymph node dissections (none, lobe-specific or extended lymph node dissection for patients without preoperative VAMLA), and patient-specific factors such as severe pleural adhesions. In our surgical center, however, we believe that these variations are part of daily practice and did not significantly vary between the surgeons nor varied over time. Fifth, our 500cc threshold for blood loss as a technique-related complication was chosen arbitrarily since the optimal intraoperative blood loss threshold for the risk of postoperative complications is still unknown, and current literature reports threshold values between 100 and 500cc [28–30]. Finally, we did not include survival data, which may be subject to future research to evaluate the effect of the learning curve and inter-surgeon differences on survival.

## CONCLUSION

In conclusion, learning of uniportal VATS for lobectomy is safe without unacceptably high complication rates for surgeons with multiportal VATS lobectomy experience, and surgery duration declined after 14 to 26 procedures. However, the number of procedures to complete the different stages of mastery remains unknown.

## Supplementary Material

ivad135_Supplementary_Data

## Data Availability

The data underlying this article will be shared on reasonable request to the corresponding author. **Iris E.W.G. Laven:** Conceptualization; Formal analysis; Methodology; Visualization; Writing—original draft. **Jean H.T. Daemen:** Conceptualization; Methodology; Validation; Writing—original draft. **Aimée J.P.M. Franssen:** Conceptualization; Methodology; Project administration; Writing—original draft. **Michiel H.M. Gronenschild:** Supervision; Writing—review & editing. **Karel W.E. Hulsewé:** Conceptualization; Methodology; Supervision; Writing—review & editing. **Yvonne L.J. Vissers:** Conceptualization; Methodology; Supervision; Writing—review & editing. **Erik R. de Loos:** Conceptualization; Methodology; Resources; Writing—review & editing. Interdisciplinary CardioVascular and Thoracic Surgery thanks Georges Decker, Haralabos Parissis, and the other anonymous reviewer(s) for their contribution to the peer review process of this article.

## ABBREVIATIONS

CUSUM: Cumulative sum
p25: 25th percentile
p75: 75th percentile
VAMLA: Video-assisted mediastinoscopic lymphadenectomy
VATS: Video-assisted thoracoscopic surgery
