# Supplementary material for: Uniportal video-assisted thoracoscopic surgery for lobectomy: the learning curve
Source: Interdiscip Cardiovasc Thorac Surg. 2023 Aug 12;37(2):ivad135. doi: 10.1093/icvts/ivad135 (PMC10469110; doi:10.1093/icvts/ivad135)
Supplement: ivad135_Supplementary_Data [file ivad135_supplementary_data.docx]

# Supplementary Tables

**Supplementary Table 1** Uniportal VATS lobectomy learning curve studies. VATS=video-assisted thoracoscopic surgery

| Author | Year | Number of patients (n) | Number of surgeons (n) | Institution or per surgeon | Prior experience | Learning curve outcome(s) | Method learning curve analysis | Initial learning phase (n) | Proficiency learning phase (n) |
| --- | --- | --- | --- | --- | --- | --- | --- | --- | --- |
| Bedetti [1] | 2017 | 73 | 4 | Institution | Basic and advanced VATS procedures | 1. Surgery duration (conversion)  2. Postoperative complications | Two groups: group 1 of the first 30 patients versus group 2 of 43 patients. Cumulative failure rates were plotted and correlations calculate with Spearman’s Rank-Order Correlation  1. CUSUM - p0=50%, p1=70%  2. CUSUM - p0=5%, p1=10% | 30 | NR |
| Li [2] | 2022 | 538 | 1 | Per surgeon | >260 multiportal VATS operations per year and 70 op-heart procedures | Surgery duration | Joinpoint linear regression analysis and unadjusted CUSUM | 52 | 156 |
| Liu [3] | 2018 | 120 | 1 | Per surgeon | Conventional VATS | Surgery duration | CUSUM. Per 30 patients CUSUM results. | 20 upper lobectomy  19 lower lobectomy | NR |
| Nachira [4] | 2018 | 43 | 2 | Institution | Open lobectomies, muscle-sparing anterolateral thoracotomy, triportal, biportal VATS, and robotic lobectomies | Surgery duration | CUSUM | 25 | NR |
| Stamenovic [5] | 2019 | 52 | 1 | Per surgeon | Minimally invasive surgery | 1. Surgery duration  2. Harvested lymph nodes | CUSUM | 27 | 39 |
| Vieria [6] | 2020 | 274 | 1 | Per surgeon | Multiportal VATS | Surgery duration | Cubic splines method | 60 | 140 |
| Wu [7] | 2020 | 356 | 8 | Per surgeon | Varying from no minimally invasive experience (self-thought) to multiportal VATS experience to limited uniportal VATS experience | Surgery duration | CUSUM | 30 | NR |
| Zhai [8] | 2022 | 103 | 1 | Per surgeon | No open lobectomies, 62 triportal VATS lobectomies and <5 uniportal VATS lobectomies | 1. Surgery duration  2. Surgical failure* | 1. CUSUM  2. RA-CUSUM | 1. 34  2. 61 | 1. 67  2. NR |

*Surgical failure encompassed conversion, intra- or postoperative complications, harvested lymph nodes <10 and 30-day readmission. CUSUM=cumulative sum.

**Supplementary Table** **2** Complications definitions.

| Complications^*^ | Definition |
| --- | --- |
| Arrhythmia (e.g., atrial fibrillation) | Postoperative onset (de novo) of atrial fibrillation or atrial flutter requiring medical treatment or cardioversion. |
| Blood transfusion | A blood transfusion within 48 hours after surgery is recorded as a complication. |
| Deep incisional surgical site infection (SSI) [9] | Infection occurring within 30 days after initial surgery that involves the deep soft tissues, appears to be related to the procedure and has at least one of the following clinical presentations:   - purulent drainage from the deep incision; - spontaneous dehiscent or deliberate opening of the wound with at least one of the following symptoms (in combination with a positive or absent culture): temperature >38 degrees, and pain or tenderness; - an abscess or other evidence of a deep incisional infection found during reoperation, on direct examination, radiologic examination, or histopathologic examination. |
| Deep venous thrombosis | A deep venous thrombosis within 30 days following initial surgery had to be confirmed by a Doppler ultrasound. |
| Empyema | Empyema was scored according to the later-mentioned “organ/space SSI”-definition. |
| Intraoperative bleeding | Intraoperative bleeding was defined as blood loss of more than 500cc due to a surgical failure. |
| Mortality | Death within 30 days following initial surgery associated with the healthcare intervention. |
| Myocardial infarct | Myocardial infarction within 30 days after initial surgery, which was evidenced by one of the following criteria:   - appearance of a new Q wave in ≥2 contiguous leads on electrocardiographic (ECG); transmural infarction; - by clinical, angiographic ECG signs (non-Q wave); subendocardial infarction; - evidence of laboratory isoenzymes related to myocardial necrosis. |
| Organ/space SSI (e.g., empyema) [10] | Infection occurring within 30 days after initial surgery, related to the operative procedure, involving any anatomy part (e.g., spaces or organs), and at least one of the following clinical presentations:   - purulent drainage from a transcutaneous drain; - a positive culture from fluid or tissue in the organ/space; - an abscess or other evidence of an organ/space confining infection found during reoperation, on direct examination, radiologic examination, or histopathologic examination. |
| Pleural effusion requiring intervention | Radiographically proven and symptomatic (e.g., dyspnea, tachypnea) pleural effusion (e.g., chylothorax) within 30 days following initial surgery requiring intervention (e.g., needle aspiration, chest drain insertion). |
| Pneumonia [11] | The presence of radiographically confirmed new lung infiltrate and at least one of the following clinical presentations indicating that the infiltrate is of an infectious origin, within 30-days of surgery:   - the onset of fever (>38.3 °C); - purulent sputum; - leukocytosis (>10,000 mm^-3^ [10 x 10/L]); - a decline in oxygenation. |

| Pneumothorax requiring intervention | Radiographically confirmed pneumothorax (>2.5 cm from the apex of the lung or lateral chest wall), occurring within 30 days following initial surgery requiring an intervention (e.g., needle aspiration, chest tube drainage, placement of a vacuum pump). |
| --- | --- |
| Prolonged air leakage | Thoracic drainage duration >5 days after surgery within 30 days following initial surgery |
| Pulmonary embolism | A pulmonary embolism within 30 days following initial surgery had to be radiographically confirmed using a chest radiograph or a computed tomography angiography. |
| Readmission | Rehospitalization within 30 days from a previous discharge associated with the performed healthcare intervention. |
| Recurrent laryngeal nerve palsy | Intraoperative permanent damage of the recurrent laryngeal nerve within 30 days after surgery with the following clinical presentations:   - ipsilateral vocal cord paralysis confirmed by an ear-nose-throat specialist at least three months postoperatively; - persistent hoarseness without improvement after speech therapy at least one year postoperatively. |
| Reoperation for bleeding | Postoperative bleeding within 30 days following initial surgery requiring reoperation. |
| Respiratory failure | Respiratory failure within 30 days following initial surgery requiring admission to the intensive care unit (ICU) with high-flow oxygen therapy (Optiflow®) or invasive mechanical ventilation. |
| Superficial incisional SSI [9] | Infection occurring within 30 days following surgery that only involves the skin or subcutaneous tissue with at least one of the following clinical presentations:   - purulent drainage; - positive culture of the superficial incision or fluid; - or at least one of the following symptoms (in combination with a positive culture or the wound being deliberately opened): pain or tenderness, localized swelling, redness, or heat. |

^*^Complications were defined as all adverse events associated with the performed healthcare intervention, unrelated to the disease within 30 days following initial surgery (3).

**Supplementary Table 3** Complications classified as technique-related or other complications.

| **Classification of complications** |
| --- |
| Technique-related complications |
| Conversion due to technical difficulty |
| Conversion due to a complication |
| Intraoperative bleeding > 500cc due to a surgical failure |
| Mortality |
| Reoperation for postoperative bleeding, empyema, prolonged air leakage, or a bronchopleural fistula |
| Drain, pleura-cath, or vacuum pump placement |
| Empyema |
| Blood transfusion from the start of surgery until two postoperative days |
| Wound infection |
| Prolonged chest tube duration (>5 days) |
| Recurrent laryngeal nerve palsy |
| Chylothorax |
| Other complications |
| Respiratory failure |
| Reintubation |
| Atelectasis |
| Bronchopneumonia |
| Thrombotic incident |
| Atrial fibrillation (de novo) |
| Congestive heart failure |
| COPD exacerbation |
| Hyperkaliemia |
| Overfilling |
| Renal impairment |
| Urinary retention |
| Urinary tract infection |
| Readmission within 30 days |

**Supplementary Table 4** Uniportal VATS lobectomy results of previously published articles with a uniportal VATS group size of 100 patients or more. VATS=video-assisted thoracoscopic surgery

| Author | Year | Patients (n) | **Mean surgery duration (min)** | Conversion | Intraoperative bleeding | Mortality | Reoperation | (re)insertion drain or pleura-cath | Postoperative bleeding | Empyema | Blood transfusion | Wound infection | Prolonged air leakage | Recurrent laryngeal nerve palsy | Chylothorax | **Technique-related complication rate** |
| --- | --- | --- | --- | --- | --- | --- | --- | --- | --- | --- | --- | --- | --- | --- | --- | --- |
| **Cohort studies** | | |  |  |  |  |  |  |  |  |  |  |  |  |  |  |
| Al-Ameri [12] | 2019 | 122 (134*) | - | 14* | - | - | - | 0 | 6 | - | - | 1 | 2 | 0 | - |  |
| Bourdages-Pageau [13] | 2020 | 247 | 137 | 11 | - | - | - | 0 | 1 | 4 | 1 | 2 | 6 | 1 | 31 |  |
| Hirai [14] | 2019 | 142 | 152 | 10 | - | - | - | 0 | - | - | - | - | - | - | - |  |
| Liu [15] | 2015 | 100 | 179 | - | - | - | - | - | - | - | - | - | - | - | - |  |
| Liu** [16] | 2019 | 166 | 89 | 5 | - | - | - | - | - | - | - | - | - | - | - |  |
| Shen [17] | 2015 | 100 | 95 | 1 | - | - | - | 0 | - | - | - | - | - | - | - |  |
| Tosi [18] | 2019 | 172 (187*) | 195 | 15 | - | - | - | - | - | - | 2 | - | - | - | 11 |  |
| Wang [19] | 2018 | 153 | 193 | - | - | - | - | - | 2 | - | - | - | - | 2 | 19 |  |
| **Learning curve studies** | | |  |  |  |  |  |  |  |  |  |  |  |  |  |  |
| Han [20] | 2017 | 203 | 189 | 11 | - | - | - | - | - | - | - | - | - | - | - |  |
| Li [2] | 2022 | 538 | 172 | 38 | - | - | 3 | 0 | - | - | - | 1 | - | 10 | 41 |  |
| Vieira [6] | 2020 | 274 | 135 | 21 | 18 | 3 | - | 0 | - | - | - | - | - | - | 37 |  |
| Wu [7] | 2020 | 356 | 198 | 19 | - | - | 17 | 2 | - | - | 2 | - | - | 2 | 37 |  |
| Zhai [8] | 2022 | 103 | 113 | 4 | - | - | - | 0 | - | - | - | - | - | - | 5 |  |
| **Complication overall percentage** | | |  |  |  |  |  |  |  |  |  |  |  |  |  |  |
| All cohort studies | | | 149 | 7.3% | - | 0.0% | 1.7% | 1.6% | 1.2% | 0.8% | 2.2% | 0.6% | 12.6% | 1.5% | 1.2% | **30.6%** |
| All learning curve studies | | | 161 | 6.3% | - | 2.2% | 0.2% | - | - | 0.6% | 0.2% | - | 1.3% | 9.4% | - | **21.0%** |
| All studies combined | | | 154 | 6.7% | 6.6% | 1.1% | 2.2% | 0.1% | 1.7% | 1.6% | 0.9% | 0.4% | 2.2% | 1.1% | 10.4% | **29.8%** |

*Conversion to multiportal video-assisted thoracoscopic surgery. Note, these patients were located in the multiportal VATS group for further analysis. **Initial 100 cases were excluded to minimize the learning curve effect. VATS=video-assisted thoracoscopic surgery.

# References

[1] Bedetti B, Bertolaccini L, Solli P, Scarci M. Learning curve and established phase for uniportal VATS lobectomies: The Papworth experience. J Thorac Dis 2017;9:138–142.

[2] Li WH, Cheng H, Gan XF, Li XJ, Wang XJ, Wu XW, et al. Learning curve of uniportal video-Assisted thoracoscopic lobectomy: An analysis of the proficiency of 538 cases from a single centre. Interact Cardiovasc Thorac Surg 2022;34:799–807.

[3] Liu X, Chen X, Shen Y, Wang H, Feng M, Tan L, et al. Learning curve for uniportal video-assisted thoracoscopic surgery lobectomy—results from 120 consecutive patients. J Thorac Dis 2018;10:5100–5107.

[4] Nachira D, Meacci E, Porziella V, Vita ML, Congedo MT, Chiappetta M, et al. Learning curve of uniportal video-assisted lobectomy: analysis of 15-month experience in a single center. J Thorac Dis 2018;10:S3662–S3669.

[5] Stamenovic D, Messerschmidt A, Schneider T. Cumulative Sum Analysis of the Learning Curve for Uniportal Video-Assisted Thoracoscopic Lobectomy and Lymphadenectomy. J Laparoendosc Adv Surg Tech 2019;29:914–920.

[6] Vieira A, Bourdages-Pageau E, Kennedy K, Ugalde PA. The learning curve on uniportal video-assisted thoracic surgery: An analysis of proficiency. J Thorac Cardiovasc Surg 2020;159:2487–2495.

[7] Wu CF, Paradela M, Wu CY, Mercedes de la T, Fernandez R, Delgado M, et al. The time course for developing competence in single port video- assisted thoracoscopic lobectomy. Medicine (Baltimore) 2020;99:e19459.

[8] Zhai R, Liu H, Wang J, Shan L, Luo M, Yao F. Extensive open lobectomy experience is not a prerequisite for learning uniportal video-assisted thoracic surgery lobectomy. J Surg Oncol 2022;126:1104–1113.

[9] Horan TC, Gaynes RP, Martone WJ, Jarvis WR, Emori TG. CDC Definitions of Nosocomial Surgical Site Infections, 1992: A Modification of CDC Definitions of Surgical Wound Infections. Infect Control Hosp Epidemiol 1992;13:606–608.

[10] Jacobs JP, Mavroudis C, Jacobs ML, Maruszewski B, Tchervenkov CI, Lacour-Gayet FG, et al. What is Operative Mortality? Defining Death in a Surgical Registry Database: A Report of the STS Congenital Database Taskforce and the Joint EACTS-STS Congenital Database Committee. Ann Thorac Surg 2006;81:1937–1941.

[11] Kalil AC, Metersky ML, Klompas M, Muscedere J, Sweeney DA, Palmer LB, et al. Management of Adults With Hospital-acquired and Ventilator-associated Pneumonia: 2016 Clinical Practice Guidelines by the Infectious Diseases Society of America and the American Thoracic Society. Clin Infect Dis 2016;63:e61–e111.

[12] Al-Ameri M, Sachs E, Sartipy U, Jackson V. Uniportal versus multiportal video-assisted thoracic surgery for lung cancer. J Thorac Dis 2019;11:5152–5161.

[13] Bourdages-Pageau E, Vieira A, Lacasse Y, Figueroa PU. Outcomes of Uniportal vs Multiportal Video-Assisted Thoracoscopic Lobectomy. Semin Thorac Cardiovasc Surg 2020;32:145–151.

[14] Hirai K, Usuda J. Uniportal video-assisted thoracic surgery reduced the occurrence of post-thoracotomy pain syndrome after lobectomy for lung cancer. J Thorac Dis 2019;11:3896–3902.

[15] Liu CC, Shih CS, Pennarun N, Cheng CT. Transition from a multiport technique to a single-port technique for lung cancer surgery: Is lymph node dissection inferior using the single-port technique? Eur J Cardiothorac Surg 2015;49:i64–i72.

[16] Liu Z, Yang R, Shao F. Comparison of Postoperative Pain and Recovery between Single-Port and Two-Port Thoracoscopic Lobectomy for Lung Cancer. Thorac Cardiovasc Surg 2019;67:142–146.

[17] Shen Y, Wang H, Feng M, Xi Y, Tan L, Wang Q. Single- versus multiple-port thoracoscopic lobectomy for lung cancer: A propensity-matched study. Eur J Cardiothorac Surg 2015;49:i48–i53.

[18] Tosi D, Nosotti M, Bonitta G, Mazzucco A, Righi I, Mendogni P, et al. Uniportal and three-portal video-assisted thoracic surgery lobectomy: Analysis of the Italian video-assisted thoracic surgery group database. Interact Cardiovasc Thorac Surg 2019;29:714–721.

[19] Wang G, Xiong R, Wu H, Xu G, Li C, Sun X, et al. Short-term outcome of uniportal and three portal video-assisted thoracic surgery for patients with non-small cell lung cancer. Chinese J Lung Cancer 2018;21:896–901.

[20] Han KN, Kim HK, Choi YH. Midterm outcomes of single port thoracoscopic surgery for major pulmonary resection. PLoS One 2017;12:e0186857.
